# Analysis of Perioperative Risk Factors for Clostridium difficile Infection After a Colectomy

**DOI:** 10.7759/cureus.20142

**Published:** 2021-12-03

**Authors:** Karen Kong, Sara S Soliman, Rolando H Rolandelli, Matthew J Elander, Joseph Flanagan, Daniel Hakakian, Zoltan H Nemeth

**Affiliations:** 1 Department of Surgery, Morristown Medical Center, Morristown, USA

**Keywords:** ileocolic resection, left hemicolectomy, intestinal microflora, oral antibiotics, terminal ileum, clostridium difficile infection

## Abstract

Introduction

The removal of the terminal ileum may interfere with gut-associated lymphoid tissue function, reduce bile salt reabsorption, and change intraluminal pH, which may contribute to the development of *Clostridium difficile* infection (CDI) after ileocolic resections. Therefore, we compared CDI incidence among patients who underwent a colectomy with or without removal of the terminal ileum.

Methods

Using the 2016 American College of Surgeons National Surgical Quality Improvement Program (ACS NSQIP) Targeted Colectomy database, we identified 17,962 patients who underwent a left-sided colectomy without removal of the terminal ileum and 5,929 patients who underwent an ileocolic resection involving the removal of the terminal ileum. Patients who underwent an emergency operation or had enterocolitis as the indication for surgery were excluded.

Results

Patients who underwent an ileocolic resection developed higher rates of postoperative CDI than those who underwent a left hemicolectomy (p<0.001). Multivariate logistic regression analysis demonstrated that removing the ileum was associated with a 50% higher risk of developing CDI than patients who underwent a left-sided colectomy. Additional risk factors for developing postoperative CDI were advanced age (p=0.001) and mechanical bowel preparation (p=0.001). On the other hand, factors independently associated with a lower risk of postoperative CDI were male gender (p<0.001), preoperative oral antibiotics (p<0.001), and preoperative chemotherapy use within 90 days (p<0.013).

Conclusion

Overall, patients who undergo operations involving the removal of the ileum are at higher risk for developing CDI. To reduce the risk among these patients, we suggest employing preoperative oral antibiotics in part of bowel preparation. Furthermore, it is critical to maintain hygienic measures, such as handwashing and disinfecting surfaces, and attentive care for these patients.

## Introduction

*Clostridium difficile* infection (CDI) is becoming more common in hospital settings, affecting approximately half a million people and accounting for nearly $1.5 billion in healthcare costs annually in the United States [1-3]. More recently, readmission rates after surgery have gained interest within the surgical community as they may be an indicator of adverse medical outcomes and surgical performance [4].

While the risk factors for developing CDI are well-documented in the general population, the risk factors for developing CDI after a colectomy are not as clear [5]. More specifically, determining the effect of different colorectal surgeries on the rate of postoperative CDI is not well-established. One study investigated the impact of a proctectomy when a total abdominal colectomy was performed but did not find a significant difference in CDI risk between the two operations [6]. Another study that utilized the American College of Surgeons National Surgical Quality Improvement Program (ACS-NSQIP) database compared elective stoma reversals with elective colectomies and found that elective stoma reversals are associated with a higher incidence of CDI [7]. Neither of these studies has investigated the clinical impact of the removal of part of the terminal ileum on the rate of CDI after a colectomy.

Based on observations made at our institution, individuals who undergo an ileocolic resection appear to be at a higher risk of developing postoperative CDI than left-sided colectomy patients. Therefore, we hypothesize that the removal of part of the terminal ileum, in addition to the right colon, will significantly change the physiology of the remaining gut in a way that may promote its colonization by *C. difficile*.

## Materials and methods

After obtaining approval for this study (project name: Risk factors and outcomes for patients who develop *C. difficile* after colectomy, number: 1280851-1) by the Atlantic Health System Institutional Review Board, we obtained the 2016 ACS NSQIP Targeted Colectomy Database and the 2016 ACS NSQIP Complete Database. We merged these two data files according to case ID numbers, which yielded a total of 35,908 patients. All patients with enterocolitis as the primary indication for surgery were excluded since we only wanted to investigate the incidence of CDI after and not before a colectomy. We also excluded emergent patients to upkeep homogeneity among the patient population and excluded external factors that can affect the rates of postoperative CDI.

Current procedural terminology (CPT) codes were used to stratify patients into an ileocolic resection group (CPT codes 44160 and 44205) and a left hemicolectomy group (CPT codes 44140, 44141, 44143, 44144, 44145, 44147, 44204, 44206, 44207, and 44208). Univariate analysis was used to identify significant differences in perioperative factors, comorbidities, and complications between the two groups. Chi-square test was used for categorical variables, and student’s t-test was utilized for continuous variables. Of note, we analyzed more complications than what is listed in this article. These complications are unplanned intubation, deep incisional surgical site infection, superficial incisional surgical site infection, acute renal failure, and ventilation > 48 hours. However, they were not mentioned as they did not reach statistical significance (p<0.05). Factors that were significant in univariate analysis were included in multivariate logistic regression analysis to determine the risk factors associated with developing postoperative CDI. All data stratification was performed using Microsoft Excel, and statistical analysis was conducted using Minitab 17.

This article was previously presented as an e-poster at the 2020 American College of Surgeons (ACS) Quality and Safety Conference and the 2019 American Society of Colon and Rectal Surgeons Annual Scientific Meeting.

## Results

Overall, our study included 23,891 patients, with 5,929 patients in the ileocecectomy group and 17,962 patients in the left hemicolectomy group. Analysis of general demographic data demonstrated significant differences between both patient populations for age and gender (Table 1). More specifically, the ileocecectomy group was older on average and had more male patients. Furthermore, this cohort also had higher rates of preoperative systemic inflammatory response syndrome (SIRS), diabetes, insulin usage, dyspnea, chronic obstructive pulmonary disease (COPD), and steroid usage (Table 1). In contrast, the left hemicolectomy group had a significantly higher rate of preoperative wound infection (1.1% vs. 0.7%, p=0.01, Table 1).

**Table 1:** Demographic factors and comorbidities SEM, standard error of mean; COPD, chronic obstructive pulmonary disorder; SIRS, systemic inflammatory response syndrome

Factors	Ileocolic eesection		Left hemicolectomy		P-value
	Count	%	Count	%	
Age ± SEM	62.0 ± 0.21		61.3 ± 0.09		0.002
Male	2,760	46.6%	8,813	49.1%	0.001
Race					
White	4,374	73.8%	13,517	75.3%	0.023
American Indian or Alaska native	9	0.2%	107	0.6%	<0.001
Asian	118	2.0%	553	3.1%	<0.001
Black or African American	611	10.3%	1,431	8.0%	<0.001
Native Hawaiian or Pacific Islander	6	0.1%	51	0.3%	0.12
Comorbidities					
Diabetes	967	16.3%	2,701	15.0%	0.018
SIRS	33	0.6%	47	0.3%	0.001
COPD	312	5.3%	737	4.1%	<0.001
Wound infection	44	0.7%	203	1.1%	0.01
Dyspnea	463	7.8%	961	5.4%	<0.001
Ascites	21	0.4%	47	0.3%	0.246
Congestive heart failure	41	0.7%	119	0.7%	0.812
Hypertension	2,868	48.4%	8,489	47.3%	0.137
Renal failure	4	0.1%	14	0.1%	0.799
Dialysis	19	0.3%	84	0.5%	0.134
Disseminated cancer	315	5.3%	988	5.5%	0.581
Smoker	913	15.4%	2,940	16.4%	0.079
Weight loss > 10% of body weight six months prior to surgery	196	3.3%	567	3.2%	0.571

In univariate analysis of perioperative factors, we found that the left hemicolectomy cohort had higher rates of mechanical bowel prep (56.7% vs. 63.6%, <0.001, Table 2) and oral antibiotic use (46.7% vs. 49.5%, p<0.001, Table 2). In contrast, the rates of steroid use for a chronic condition and steroid or immunosuppressant use for IBD were significantly higher among patients who underwent an ileocolic resection (p<0.001, Table 2). Furthermore, patients who underwent an ileocolic resection had longer operation times (153.1 ± 0.98 minutes vs. 199 ± 0.72 minutes, p<0.001, Table 2). Although, there were no differences noted in the average length of stay. Post-operatively, the incidence of CDI was higher among patients who underwent an ileocolic resection than patients who underwent a left hemicolectomy (1.8% vs. 1.2%, p<0.001, Table 2). Not only this, but rates of postoperative myocardial infarction, bleeding requiring transfusion, deep vein thrombosis (DVT), and septic shock were also higher among patients who underwent an ileocolic resection (Table 2). The only complication that was more prevalent among patients who underwent a left hemicolectomy was urinary tract infection (1.3% vs. 1.7%, p=0.021, Table 2).

**Table 2:** Perioperative factors and complications SEM, standard error of mean; CDI, *Clostridium difficile* infection; IBD, inflammatory bowel disease; SSI, surgical site infection; CVA, cerebrovascular accident; CPR, cardiopulmonary resuscitation; DVT, deep vein thrombosis; OR, operating room.

Factor	Ileocolic resection		Left hemicolectomy		P-value
	Count	%	Count	%	
Preoperative mechanical bowel prep	3,363	56.7%	11,422	63.6%	<0.001
Preoperative oral antibiotic prep	2,771	46.7%	8,892	49.5%	<0.001
Operation time ± SEM	153.1 ± 0.98		199 ± 0.72		<0.001
Length of Stay ± SEM	5.3 ± 0.08		5.3 ± 0.04		0.53
Steroid use for a chronic condition	794	13.4%	804	4.5%	<0.001
Steroid/Immunosuppressant for IBD	703	11.9%	503	2.8%	<0.001
Chemotherapy	154	2.60%	1,537	8.56%	<0.001
Complications					
CDI	106	1.8%	210	1.2%	<0.001
Urinary tract infection	77	1.3%	312	1.7%	0.021
Septic shock	59	1.0%	124	0.7%	0.02
Myocardial infarction	43	0.7%	75	0.4%	0.003
Bleeding requiring transfusion	401	6.8%	969	5.4%	<0.001
DVT requiring therapy	63	1.1%	142	0.8%	0.049
Organ/space SSI	216	3.6%	605	3.4%	0.314
Wound disruption	30	0.5%	104	0.6%	0.514
Pneumonia	94	1.6%	233	1.3%	0.098
Pulmonary embolism	21	0.4%	75	0.4%	0.504
Progressive renal insufficiency	30	0.5%	109	0.6%	0.376
Stroke/CVA	12	0.2%	26	0.1%	0.334
Cardiac arrest requiring CPR	22	0.4%	54	0.3%	0.404
Sepsis	124	2.1%	341	1.9%	0.351
Return to OR	209	3.5%	676	3.8%	0.399

In multivariate logistic regression analysis, we sought to identify factors independently associated with developing postoperative CDI. Interestingly, we found that removal of the ileum was associated with a 50% higher risk of CDI as opposed to the removal of the left colon (OR, 1.52 [95% CI (1.18, 1.85)], p=0.001, Table 3). Additional risk factors independently associated with developing CDI were mechanical bowel prep and advanced age (p<0.05). On the other hand, undergoing chemotherapy, oral antibiotic bowel prep, and male gender were associated with about 50%, 40%, and 33% lower risk of CDI, respectively (p<0.05).

**Table 3:** Multivariate logistic regression analysis for CDI CDI, *Clostridium difficile* infection; COPD, chronic obstructive pulmonary disorder; SIRS, systemic inflammatory response syndrome; IBD, inflammatory bowel disease; CI, confidence interval.

Factor	Odds ratio (95% CI)	P-value
Age	1.0148 (1.0061, 1.0235)	0.001
Operation time	1.0016 (1.0004, 1.0027)	0.011
Ileocolic resection	1.5201 (1.1855, 1.9491)	0.001
Male	0.6628 (0.5262, 0.8348)	<0.001
Diabetes	0.7321 (0.5198, 1.0310)	0.064
Dyspnea	0.9511 (0.6030, 1.5000)	0.828
COPD	1.4316 (0.8986, 2.2807)	0.147
Wound infection	2.1718 (1.0063, 4.6870)	0.076
Steroid use for a chronic condition	1.1951 (0.6791, 2.1034)	0.542
Steroid/Immunosuppressant for IBD	1.6625 (0.8828, 3.1310)	0.118
Weight loss	1.2692 (0.7222, 2.2303)	0.423
Chemotherapy	0.4930 (0.2665, 0.9120)	0.013
Preoperative mechanical bowel prep	1.5807 (1.2164, 2.0541)	0.001
Preoperative oral antibiotic prep	0.6107 (0.4745, 0.7860)	<0.001
White	1.1670 (0.8922, 1.5266)	0.253
SIRS	2.3947 (0.9659, 5.9370)	0.095

## Discussion

Using the ACS NSQIP Targeted Colectomy database, we found that patients who underwent a partial colectomy with the removal of part of the terminal ileum were associated with having a higher risk of developing CDI postoperatively. The small bowel is known to be the only location of important gastrointestinal (GI) immune organ Peyer’s patches, and their concentration is exceptionally high in the terminal ileum, where about 46% of them are located [8,9]. Peyer’s patches are essential in mucosal immune responses as they monitor the intestinal microflora and prevent the proliferation of pathogenic bacteria [9]. Thus, removing even a part of the terminal ileum may affect a line of immunologic defense and change the immune response within the GI tract. This can potentially cause an imbalance in microflora and may lead to a higher risk of CDI.

Another critical function of the terminal ileum is the reabsorption of bile salts. Typically, the terminal ileum reabsorbs 95% of bile salts and transports them back to the liver [10]. Bile salts are known to be a factor in allowing *C. difficile* spores to germinate. Therefore, the removal of part of the terminal ileum can results in excess bile salts in the colon and a higher risk of CDI [11].

Anatomically, a partial colectomy with the removal of the terminal ileum inherently means the removal of the cecum, which may also contribute to increasing the risk of CDI. The cecum is acidic, with a pH of approximately 5.7, from producing fatty acids through fermentation [12,13]. Studies have suggested that an alkaline colonic environment predisposes patients to CDI. Therefore, the removal of the acidic cecum along with the terminal ileum makes the colon more alkaline, potentially increasing the risk of CDI [14].

Furthermore, advanced age and mechanical bowel prep were also independent risk factors for developing CDI. At the same time, pre-operative oral antibiotic use and male gender were independently associated with lower risks of CDI. Previous literature has even iterated that advanced age is a risk factor for CDI [15] as it is correlated with more comorbidities and weaker immune responses towards pathogens of the GI tract. The literature has also stated that pre-operative oral antibiotic use either had no effect or lowered the risk of postoperative CDI [16,17]. A prospective study analyzing the impact of oral metronidazole as pre-operative bowel prep found that it also reduced the rate of CDI [17,18]. This antibiotic is usually utilized to treat colitis so that the pre-operative use may reduce its colonization by *C. difficile* [18]. Overall, if it is deemed necessary to remove part of the terminal ileum, the physician should prepare for CDI while the patient recovers. We recommend implementing oral antibiotics in part of pre-operative bowel preparation for patients undergoing the right colectomies to reduce CDI risk. Other precautions can be taken, such as discontinuing antibiotics postoperatively and increasing hygienic measures, such as regular disinfection of high-risk surfaces and better hand hygiene. However, before this, it may be helpful to initiate research analyzing which antibiotic is associated with better outcomes as their effectiveness against postoperative CDI may differ.

Additionally, chemotherapy within 90 days of surgery was associated with having a protective effect against developing a post-colectomy CDI in multivariate analysis. A previous study found that chemotherapy patients’ risk for CDI depended on the type of cancer they had [19]. It was found in a retrospective study of 225 patients that patients with GI tumors were less prone to CDI [19]. This could be explained by the chemotherapy targeting mucosal cells, which would lead to faster shedding of the mucosal layer of the gut that *C. difficile* would typically colonize.

While the ACS NSQIP database provides a large sample of patients to analyze, there are limitations. The nature of the database restricts us from making definitive conclusions about the exact cellular mechanisms of how the removal of the terminal ileum is associated with higher rates of CDI. Another limitation is the lack of details regarding the impact of removing the right colon on gut peristalsis. Since removing the terminal ileum means removing the ileocecal valve, patients with ileocecal resections are more likely to have diarrhea than left-sided colectomy patients. A higher incidence of diarrhea in these patients usually means a higher testing rate for CDI, leading to more positive cases. Therefore, this can lead to a bias in the CDI rate among patients undergoing an ileocolic resection.

## Conclusions

Our novel findings regarding the impact of the removal of the terminal ileum on developing CDI are not well-documented in the literature, and there are many reasons why its removal may be associated with CDI. For instance, the partial or total removal of Peyer’s patches, the lack of significant reduction of bile salt reabsorption, and changes in intraluminal pH may contribute to a higher rate of CDI. Despite this, efforts to reduce post-operative CDI should be continued. We suggest employing oral antibiotics in part of bowel preparation, maintaining hygienic measures, such as disinfecting high-risk surfaces and handwashing, as well as vigilant care for these patients.
